# Non-Uniform Sample Assignment in Training Set Improving Recognition of Hand Gestures Dominated with Similar Muscle Activities

**DOI:** 10.3389/fnbot.2018.00003

**Published:** 2018-02-12

**Authors:** Yao Zhang, Yanjian Liao, Xiaoying Wu, Lin Chen, Qiliang Xiong, Zhixian Gao, Xiaolin Zheng, Guanglin Li, Wensheng Hou

**Affiliations:** ^1^Key Laboratory of Biorheological Science and Technology, Ministry of Education, Bioengineering College, Chongqing University, Chongqing, China; ^2^Chongqing Engineering Research Center of Medical Electronics Technology, Chongqing, China; ^3^Key Laboratory of Human-Machine Intelligence-Synergy Systems, Shenzhen Institutes of Advanced Technology, Chinese Academy of Sciences, Shenzhen, China

**Keywords:** myoelectric control, training set, similar hand gestures, sample proportion, pattern recognition

## Abstract

So far, little is known how the sample assignment of surface electromyogram (sEMG) features in training set influences the recognition efficiency of hand gesture, and the aim of this study is to explore the impact of different sample arrangements in training set on the classification of hand gestures dominated with similar muscle activation patterns. Seven right-handed healthy subjects (24.2 ± 1.2 years) were recruited to perform similar grasping tasks (fist, spherical, and cylindrical grasping) and similar pinch tasks (finger, key, and tape pinch). Each task was sustained for 4 s and followed by a 5-s rest interval to avoid fatigue, and the procedure was repeated 60 times for every task. sEMG were recorded from six forearm hand muscles during grasping or pinch tasks, and 4-s sEMG from each channel was segmented for empirical mode decomposition analysis trial by trial. The muscle activity was quantified with zero crossing (ZC) and Wilson amplitude (WAMP) of the first four resulting intrinsic mode function. Thereafter, a sEMG feature vector was constructed with the ZC and WAMP of each channel sEMG, and a classifier combined with support vector machine and genetic algorithm was used for hand gesture recognition. The sample number for each hand gesture was designed to be rearranged according to different sample proportion in training set, and corresponding recognition rate was calculated to evaluate the effect of sample assignment change on gesture classification. Either for similar grasping or pinch tasks, the sample assignment change in training set affected the overall recognition rate of candidate hand gesture. Compare to conventional results with uniformly assigned training samples, the recognition rate of similar pinch gestures was significantly improved when the sample of finger-, key-, and tape-pinch gesture were assigned as 60, 20, and 20%, respectively. Similarly, the recognition rate of similar grasping gestures also rose when the sample proportion of fist, spherical, and cylindrical grasping was 40, 30, and 30%, respectively. Our results suggested that the recognition rate of hand gestures can be regulated by change sample arrangement in training set, which can be potentially used to improve fine-gesture recognition for myoelectric robotic hand exoskeleton control.

## Introduction

Myoelectric control systems have been widely used to control assistive and rehabilitation devices, i.e., EMG-controlled robotic hand exoskeleton (Leonardis et al., 2015), which collected the surface electromyogram (sEMG) from the forearm muscles of non-paretic hand controlling the movement of exoskeleton, and to train and/or guide the grasping or pinch task conduction of paretic hand as well. Feature classification of sEMG in time and/or frequency domain is usually employed for recognizing non-paretic hand gesture under the following principle: different hand motions/gestures are dominated with different muscle activity patterns, which result in a distinguishable sEMG feature vector (Lima et al., 2016). Although a variety of myoelectric pattern identification strategies have been proposed to classify the sEMG signals for different hand gestures, very little attention has been paid to the recognition of hand gestures dominated with similar hand muscle activity patterns (AbdelMaseeh et al., 2016). Improving the classification and identification of similar hand gestures is helpful for exquisite myoelectric control system development (Amsuess et al., 2015).

Up to date, increased interests have been focused on hand gesture recognition based on sEMG features, and high classification accuracies can be obtained (Khezri and Jahed, 2007). Usually, hand gestures dominated by different hand muscle contractions (Young et al., 2012), such as palm extension and closure, wrist flexion and extension, and supination and pronation, are used to test the classification efficiency. Therefore, it is believed that high recognition rates of the hand gesture strongly depend on differentiation of EMG activities among these hand motions. Urwyler et al. (2015) reported that a high classification accuracy (above 95%) for classifying the four or six movements. Peerdeman et al. (2011) improved the classification rate in daily hand movements by optimizing the sEMG feature sets and classification algorithm. Although numerous studies have focused on the most suitable signal feature selection and classification strategy design (Sapsanis et al., 2013), little efforts has been put to the specific demand of similar gesture recognition. As one of the most dexterous organs in the world, our hand can perform a variety of hand motions with different finger coordination patterns, and part of these hand motions are controlled with almost same hand muscle contraction patterns, such as hand pinch and hand tripod gestures. Unfortunately, these hand gestures with similar muscle activities patterns were usually excluded from hand motion classification studies due to their low sensitivity and poor classification performance (Castro et al., 2015). According to our previous work, the accuracy rate of similar gestures recognition for pinching different items or grasping bottles with different weights was less than 80% (Zhang et al., 2016). However, to train the paretic hand after stroke with a robotic hand exoskeleton, it is necessary to identify gestures with high similarity based on sEMG features detection from contralateral non-paretic hand.

The key obstacle for similar hand gesture recognition is that these hand movements are dominated with the same hand muscle’s contraction patterns (Liu et al., 2014). However, a critical factor for gesture classification is that the feature vector of different gestures should contain sufficient sensitivity and specificity (Chen et al., 2016b). In other words, the distance between gesture classes in the myoelectric feature space must be sufficiently wide. Unfortunately, distances between classes of similar gestures are diminished due to the feature vector extracted from similar muscle activation pattern are difficult to be distinguished, which deteriorate the final classification performance. In addition to the feature selection and classification algorithm optimization, the performance of hand gesture recognition highly depends on the quality of a training set (Lorrain et al., 2011). Growing evidences have shown that the design of training sample assignment, both the sample size and proportion in training set, can impact the classification accuracy. Foody et al. (1995) verified that variations in the size of each class in the training set affected the pattern of class allocation; Chen et al. (2009) demonstrated that better performance of a classifier could be achieved when optimizing a training set by expanding the sample size. Wigdahl et al. (2013a) showed that a small training set size could achieve better overall classification results when they varied the number of normal controls in corresponding training set. Generally, the sample size and proportion of the training sample play a non-ignorable role on the classification efficiency, and better classification could be obtained by optimizing the constitution of the training set (Fratini et al., 2015). Therefore, it can be presumed that optimizing the myoelectric training set could impact similar gesture recognition performance accordingly.

Due to the principle of inter-limb coordination (Luft et al., 2014), voluntary movement of non-paretic hand controlling the paretic hand activities, or bimanual training, is a promising approach for stroke rehabilitation (Oujamaa et al., 2009; Cauraugh et al., 2010). To accurately control the movement of hand exoskeleton for paretic hand training, it is essential to detect the dexterous hand motions performed by finger coordination patterns, which sometimes may be controlled with very similar hand muscle contractions. This study is to investigate how sample arrangement in training set affects the hand gesture classification accuracy. sEMG signals have been recorded from forearm hand muscles when conducting similar grasping gestures or similar pinch gestures, and the impact of the sample proportion in the training set on the recognition efficiency of similar hand gestures are evaluated by changing the sample number of each candidate gesture.

## Materials and Methods

### Participants

The protocol of this study was approved by Institutional Review Board of Shenzhen Institutes of Advanced Technology, Chinese Academy of Sciences. Seven healthy subjects (aged 24.2 ± 1.2 years, six males and one female, all right handed, height: 1.71 ± 0.17 m, and weight: 65.62 ± 8.1 kg) without neurological or muscular disease participated in this study. Explanation of the experiment and protocol were provided to all participants. Written informed consents and permission for publication of photographs for scientific and educational purposes were obtained before procedure.

### Data Acquisition

**Figure 1:**
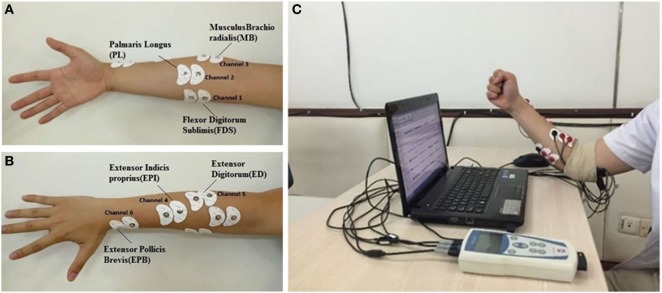
Placement of disposable surface electrodes on the forearm for surface electromyogram (sEMG) collection: **(A)** ch1(FDS), ch2(PL), and ch3(MB); **(B)** ch4(EPI), ch5(ED), and ch6(EPB); **(C)** sEMG recording during fist-grasping movement.

The sEMG signals were recorded using a surface EMG system (ME6000, Mega Electronics Ltd., Finland). Pairs of disposable surface electrodes were placed on the six forearm hand muscles: (1) the extensor pollicis brevis (EPB), (2) extensor indicis propirus (EPI), (3) flexor digitorum sublimis (FDS), (4) palmaris longus (PL), (5) musculus brachioradialis (MB), and (6) extensor digitorum (ED) (Figures 1A,B). To minimize movement artifacts, preamplified EMG sensor units are attached to the limbs using elastic gauze. The recording system bandwidth is 15–500 Hz, and the sampling rate is 1 kHz for sEMG collection.

### Experiment Protocol

**Figure 2:**
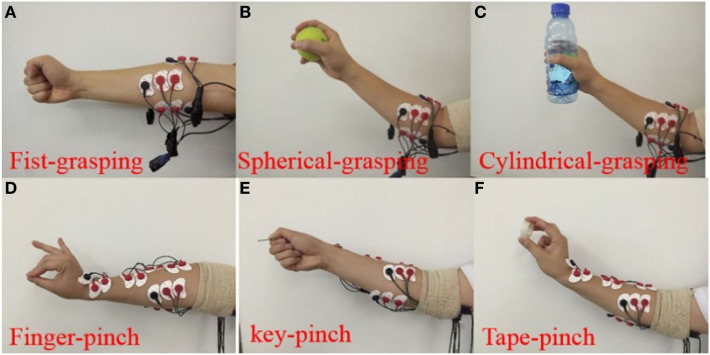
Six hand gestures were assigned for two groups of similar gestures. Grasping group includes fist-, spherical-, and cylindrical-grasping gesture **(A–C)**, Pinch group includes finger-, key-, tape-pinch gesture **(D–F)**.

Subjects are required to sits on a chair with their upper limbs vertically relaxed in the sagittal plane and forearms flexed to 90°, as shown in Figure 1C. To study desirable hand movements in daily life (Windrich et al., 2016), two sets of similar hand grasping gestures (i.e., fist, spherical, and cylindrical grasping) and pinch gestures (finger, key, and tape pinch) are conducted with the right hand (Figure 2). Verbal and visual cues are given to the participants to perform the designed movements. Each task is sustained for 4 s and followed by a 5-s rest interval to avoid fatigue. The procedure is repeated 60 times for each task, and each subject conducts a total of 180 trials for grasping and pinch movements.

### Data Analysis

#### Pre-process

We analyzed the data off-line with a customized Matlab program (the Mathworks, Natick, MA, USA). The recorded sEMG signals were bandpass filtered through a Butterworth digital filter (10–400 Hz, fourth order and zero phase) and followed by a 50-Hz digital notch filter for overcoming the power interference. Furthermore, within a 256-ms sliding window, the average IEMG (integrate sEMG) (Phinyomark et al., 2012) value was calculated as

(1) $$X_{\mathrm{iemg}}=\frac{1}{256}\sum_{i=0}^{255}\left|x(i)\right|$$

where *x*(*i*) was the *i*th sampled sEMG signal, and *X_iemg_* was the IEMG value within 256-ms time window. Once that value exceeded a predefined threshold, the muscle was activated for grasping or pinch movement. Then, the next 4-s sEMG signals was segmented into 256-ms analysis windows with an overlap of 50 ms for further processing.

#### sEMG Feature Vector Construction

**Figure 3:**
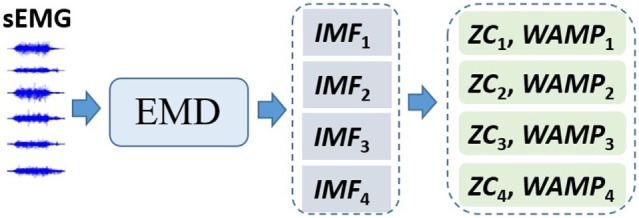
The flow diagram for the surface electromyogram (sEMG) feature vector construction.

The segmented second sEMG signal of a grasping trial or pinch trial was processed as following flow diagram (Figure 3) to construct the feature vector for hand gesture recognition.

**Figure 4:**
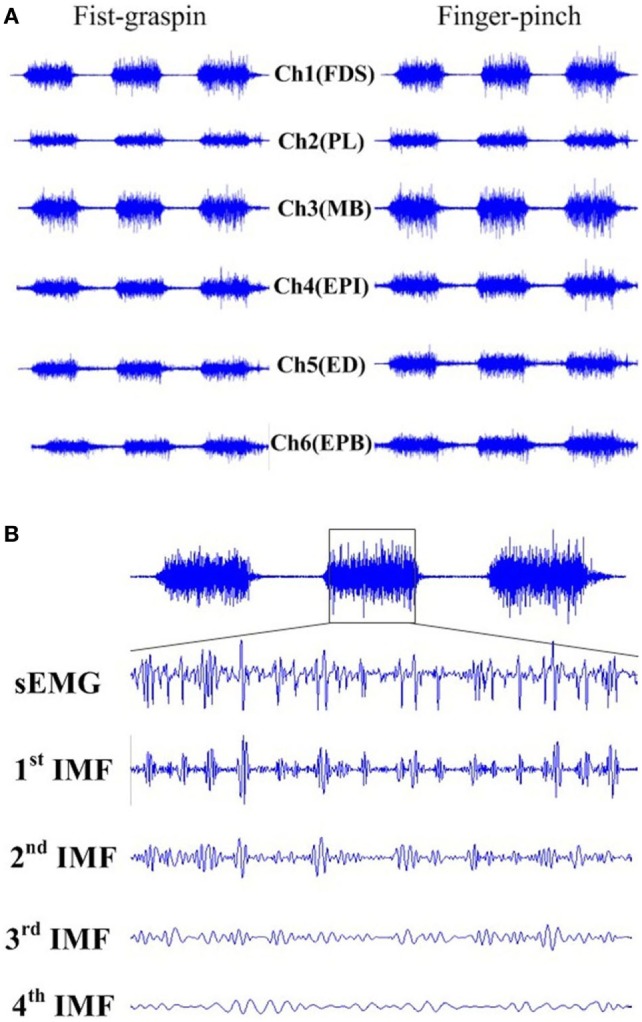
Surface electromyogram (sEMG) activities recorded from right FDS(Ch1), PL(Ch2), MB(Ch3), EPI(Ch4), ED(Ch5), and EPB(Ch6) during fist-grasping or finger-pinch **(A)**. sEMG signal segment collected from right FDS(Ch1) and its first four intrinsic mode functions for a fist-grasping trial **(B)** ED, extensor digitorum; MB, musculus brachioradialis; EPB, extensor pollicis brevis; FDS, flexor digitorum sublimis; EPI, extensor indicis propirus.

The empirical mode decomposition (EMD) (Shang et al., 2011; Hong et al., 2016) was employed to extract multichannel-recorded sEMG features for pattern recognition. In each trial, a 4-second-recorded sEMG was extracted per channel, and the EMD was used to decompose the sEMG into eight intrinsic mode functions (IMFs) as

(2) $$\text{sEMG}=\sum_{i=1}^{8}\;\text{IM}{\text{F}}_{i}+r$$

where *r* is the residual component, means the central tendency of sEMG signal. An example of sEMG segment during a fist-grasping trial and its first four IMFs is illustrated in Figure 4.

To quantify the sEMG intensity, zero crossing (ZC) (Jain et al., 2000) and Wilson amplitude (WAMP) (Castro et al., 2014) were computed for each IMF component with a window of 2 s, channel by channel. The WAMP value for the resulted sEMG IMF within 2-s window was calculated as

(3) $$\text{WAMP}=\sum_{k=2}^{2000}\;\left|\text{sEMG}\left(k\right)-\text{sEMG}\left(k-1\right)\right|$$

To reduce the dimension of the feature set, principle component analysis method (Francini et al., 2017) was applied to select the IMFs with more contributions. Here, the first four IMFs components were selected as their contribution ratio was above 90% (or the cumulative percent was 90%). As a result, the dimension of the sEMG feature vector could be reduced to eight (2 features × 4 IMFs) for one channel, and a total of 48 sEMG features were extracted for each trial.

(4) $$\begin{array}{l} A_{j}=\left[\text{Z}{\text{C}}_{1j},\text{WAM}{\text{P}}_{1j}\text{, Z}{\text{C}}_{2j},\text{WAM}{\text{P}}_{2j},\text{Z}{\text{C}}_{3j},\text{WAM}{\text{P}}_{3j},\right.\left.\text{Z}{\text{C}}_{4j},\text{WAM}{\text{P}}_{4j}\right] \end{array}$$

(5) $$\begin{array}{l} B=\left[A1,A2,\ldots ,A6\right] \end{array}$$

where *A_j_* is the feature vector of the *j*th channel (*j* = 1, … , 6), and *B* is the myoelectric feature matrix of a gesture including a six-channel sEMG feature.

#### Assessing the Recognition Efficiency with Different Sample Proportions in the Training Set

As mentioned above, 60 samples of sEMG feature vector were extracted for each hand gesture in grasping group or pinch group. For every grasping gesture or pinch gesture, 70% of 60 samples of sEMG feature (42 samples) have been randomly selected as candidate training samples, whereas the rest 30% of 60 of sEMG feature samples (18 samples) constituted the testing set. A modified classifier combining the support vector machine (SVM) (Alba et al., 2007) and genetic algorithm (GA) (Li et al., 2017; Serdio et al., 2017) was employed for its low computation cost (Marchetti et al., 2013; Martins et al., 2014). In briefly, a GA-modified SVM classifier used the sEMG feature vector (B = [A1, A2, … , A6]) as training sample for hand gesture recognition. GA was employed to filter and optimize SVM penalty coefficient (*c*) and kernel parameter (*g*), with a maximum generation of 100. Thereafter, the optimized SVM classifier with fivefold cross-validation is applied to classify hand gestures and evaluate solutions.

To assess the effect of the sample proportion of similar hand gestures on the recognition rate, we constructed a training set with a constant size of 52 samples for the grasping group or pinch group, while the sample number of each gesture was adjusted to alter the sample proportion in training set. When studying how the sample proportion of a grasping gesture or pinch gesture affects the classification rate, we step by step increased the sample number of this gesture and decreased the sample number of other two gestures to maintain a constant size for the training set. As an example shown in Table 1, when inspecting the fist-grasping gesture, we increased the sample proportion of fist-grasping from ~10% (6 samples out of 52) to ~80% (42 sample) in the grasping group, and sample proportions of spherical and cylindrical grasping decreased from ~45% (23 samples) to ~10% (5 samples). Overall, the training set always maintained a constant size of 52 samples; meanwhile, the testing set maintained a size of 54 samples (18 samples for fist, spherical, or cylindrical grasping each). The overall recognition rate of three gestures was also calculated in the testing set of grasping group (fist, spherical, and cylindrical grasping). A similar evaluation procedure was applied to spherical or cylindrical grasping in grasping group, and finger, key, or tape pinch in pinch gesture group.

**Table 1:** Example of sample number and proportion assignment in the training set and testing set for the fist grasping.

Gesture	Number of sample (sample proportion)								
	Training set								Testing set
Fist grasping	6 (11.5%)	10 (19.2%)	16 (30.8%)	20 (38.5%)	26 (50%)	30 (57.7%)	36 (69.2%)	42 (80.1%)	18 (33.3%)
Spherical grasping	23 (44.2%)	21 (40.4%)	18 (34.6%)	16 (30.8%)	13 (25%)	11 (21.2%)	8 (15.4%)	5 (9.6%)	18 (33.3%)
Cylindrical grasping	23 (44.2%)	21 (40.4%)	18 (34.6%)	16 (30.8%)	13 (25%)	11 (21.2%)	8 (15.4%)	5 (9.6%)	18 (33.3%)
Total samples	52	52	52	52	52	52	52	52	54

#### Measurement of the Feature Space Distance among Similar Hand Gestures

The Mahalanobis distance (Al-Angari et al., 2016) was used to quantify the changes in the feature space between similar hand gestures. The distance between classes (*D*_out_) is defined to measure the distance between classes of different motions by

$$\begin{array}{l} D_{\text{out}\left(i\right)}=\frac{1}{3}\sum_{i=1}^{3}\left(\text{mi}{\text{n}}_{\begin{array}{c} j=1,2,3;\\ j\neq i \end{array}}\frac{1}{2}\right.\left.\times \sqrt{{\left(\mu_{i}-\mu_{j}\right)}^{T}{\left[\frac{1}{2}\left(\sum i+\sum j\right)\right]}^{-1}\left(\mu_{i}-\mu_{j}\right)}\right) \end{array}$$

where μ*_i_* is the centroid of motion *i*, μ*_j_* is the centroid of motion *j*, and Σ*i* and Σ*j* are their covariances. The equation is used to calculate the minimum of the Mahalanobis distance between different motions. A smaller *D*_out_ indicates a shorter distance between classes of different motions. For each sample proportion in grasping training set or pinch training set, we calculate the *D*_out_ between the sEMG feature vectors of any two similar hand gestures comparing the inter-class distance.

## Results

### Recognition Rate of a Hand Gesture Increased with Its Proportion in the Training Set

**Figure 5:**
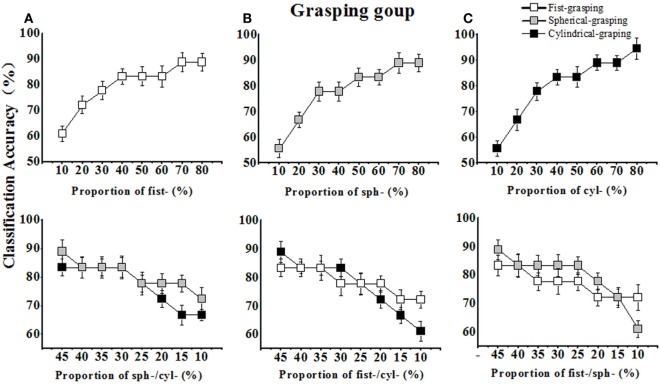
The classification accuracies (Acc.) of a grasping gesture increased with the sample proportion increasing and decreased with sample proportion decreasing in the training set. **(A)** The Acc. of fist-grasping increased with its sample proportion increasing in training set (upper), and the Acc. of spherical-/cylindrical-grasping decreased with their sample proportion decreasing in training set (lower); **(B)** The Acc. of spherical-grasping increased with its sample proportion increasing in training set (upper), and the Acc. of fist-/cylindrical-grasping decreased with their sample proportion decreasing in training set (lower); **(C)** The Acc. of cylindrical-grasping increased with its sample proportion increasing in training set (upper), and the Acc. of fist-/spherical-grasping decreased with their sample proportion decreasing in training set (lower).

In both task (grasping and pinch gesture) groups, we tested the impact of sample proportion of specific hand gesture of interest in training set on its corresponding classification performance. The proportion of a gesture of interest increased from ~10 to ~80%, while the proportion of two other gestures decreased from ~45 to ~10% as the training set maintained a constant size of 52 samples (Table 1). As shown in Table 2 and Figure 5A, the recognition rate or classification accuracy (Acc.) of the fist-grasping gesture increased from 61.1 to 88.9% when its sample number increased from 6 to 42. On other hand, when the sample number for spherical- and cylindrical-grasping gestures decreased from 23 to 5, and the recognition rates for spherical and cylindrical grasping decreased to 72.2 and 66.7%, respectively (Figure 5A, lower part). Also, as illustrated in Figures 5B,C, the recognition rate of spherical and cylindrical grasping exhibited similar trend when the sample number was adjusted step by step.

**Table 2:** The sample proportion for fist, spherical, and cylindrical grasping and the corresponding classification accuracies (mean ± SD).

Fist grasping		Spherical grasping		Cylindrical grasping		Overall Acc. (%)
Sample proportion	Acc. (%)	Sample proportion	Acc. (%)	Sample proportion	Acc. (%)	
6/52	61.1 ± 1.5	23/52	83.3 ± 1.5	23/52	88.9 ± 1.7	77.8 ± 2.8
10/52	72.2 ± 0.8	21/52	83.3 ± 1.3	21/52	83.3 ± 1.8	79 ± 2.1
16/52	77.8 ± 1.4	18/52	83.3 ± 1.4	18/52	83.3 ± 2.1	79.6 ± 1.7
20/52	83.3 ± 0.7	16/52	83.3 ± 1.2	16/52	83.3 ± 2.4	83.3 ± 2.2
26/52	83.3 ± 1.4	13/52	77.9 ± 0.8	13/52	77.8 ± 1.9	81.4 ± 2.5
30/52	83.3 ± 1.2	11/52	72.2 ± 1.9	11/52	77.8 ± 1.5	78 ± 1.9
36/52	88.9 ± 1.3	8/52	66.7 ± 1.3	8/52	77.8 ± 2.1	77.5 ± 2.2
42/52	88.9 ± 1.9	5/52	66.7 ± 1.9	5/52	72.2 ± 2.5	76 ± 2.6
17/51	79.3 ± 2.5	17/51	80.2 ± 1.6	17/51	82.3 ± 1.9	80.8 ± 2.5

**Figure 6:**
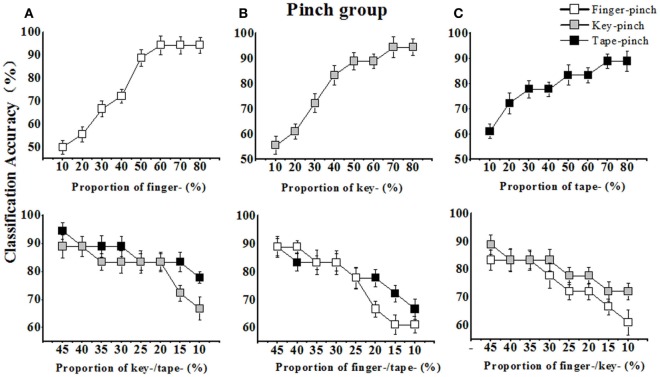
The classification accuracies (Acc.) of pinch gesture increased with the enlarged sample proportion and decreased with the dropped sample proportion in the training set. **(A)** The Acc. of finger-pinch increased with its sample proportion increasing in training set (upper), and the Acc. of key-/tape-pinch decreased with their sample proportion decreasing in training set (lower); **(B)** The Acc. of key-pinch increased with its sample proportion increasing in training set (upper), and the Acc. of finger-/tape-pinch decreased with their sample proportion decreasing in training set (lower); **(C)** The Acc. of tape-pinch increased with its sample proportion increasing in training set (upper), and the Acc. of finger-/key-pinch decreased with their sample proportion decreasing in training set (lower).

For the pinch gesture group (see Figure 6), the impact of the sample proportion for specific pinch gesture of interest on its corresponding recognition rate was similar to that for the grasping-gesture group. As listed in Table 3, when increasing the sEMG feature sample number of the finger-pinch gesture from 6 to 42, the recognition rate or classification accuracy (Acc.) of pinch increased from 50 to 94.4% (see Figure 6A). On other hand, the sample number for key- and tape-pinching gestures decreased from 23 to 5, and the corresponding recognition rates decreased to 66.7 and 77.8%, respectively [(Figure 6A), lower part].

**Table 3:** The sample proportion for finger, key, and tape-pinch and classification accuracies (mean ± SD).

Finger pinch		Key pinch		Tape pinch		Overall Acc. (%)
Sample proportion	Acc. (%)	Sample proportion	Acc. (%)	Sample proportion	Acc. (%)	
6/52	50 ± 1.8	23/52	88.9 ± 3.1	23/52	94.4 ± 2.3	75.9 ± 2.1
10/52	55.6 ± 2.1	21/52	88.9 ± 1.8	21/52	88.9 ± 1.4	77.8 ± 1.8
16/52	66.7 ± 2.2	18/52	83.3 ± 2.5	18/52	88.9 ± 2.2	79.6 ± 1.6
20/52	72.2 ± 1.9	16/52	83.3 ± 2.4	16/52	88.9 ± 1.6	81.5 ± 2.8
26/52	88.9 ± 1.5	13/52	83.3 ± 1.9	13/52	83.3 ± 2.6	85.1 ± 2.2
30/52	94.4 ± 1.4	11/52	83.3 ± 2.1	11/52	83.3 ± 2.7	87 ± 2.7
36/52	94.4 ± 2.6	8/52	72.2 ± 1.7	8/52	83.3 ± 1.9	83.3 ± 3.3
42/52	94.4 ± 3	5/52	66.7 ± 2.2	5/52	77.8 ± 2.2	79.6 ± 1.8
17/51	70.5 ± 2.2	17/51	80.6 ± 2.9	17/51	80.1 ± 1.8	79.8 ± 2.1

### Optimizing the Sample Proportion in the Training Set Improving Classification Performance of Similar Hand Gestures

**Figure 7:**
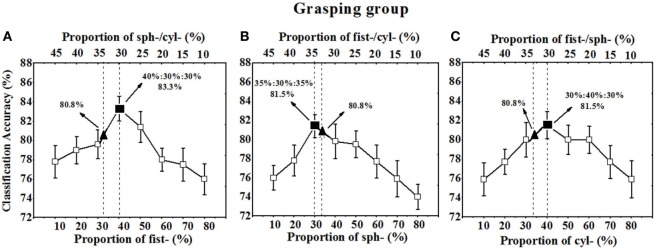
The total recognition rates of fist-, spherical-, and cylindrical-grasping gesture varied with the sample proportion. **(A)** The overall Acc. varied with the sample proportion of fist grasping; **(B)** the overall Acc. varied with the sample proportion of spherical grasping; **(C)** the overall Acc. varied with the sample proportion of cylindrical grasping; ▴ indicating the Acc. when sample proportion of fist-, spherical-, and cylindrical-grasping gesture were one third; ■ indicating the peak Acc. when sample proportion was optimized in grasping training set.

As illustrated in Figure 7A, with increasing the sample proportion for fist-grasping from ~10 to ~80% and decreasing the sample proportion for spherical or cylindrical grasping from ~45 to ~10%, the overall recognition rate of three grasping gestures increased at first and then decreased. The peak recognition rate reached 83.3% when the sample proportions for fist, spherical, and cylindrical grasping were ~40, ~30, and ~30%, respectively. Similarly, when we adjusted the sample proportion for spherical-grasping or cylindrical-grasping from ~10 to ~80%, the overall recognition rate also increased first and then finally decreased. The peak recognition rate (81.5%) occurred when the sample proportions for fist, spherical, and cylindrical grasping are ~35, ~30, and ~35%, respectively (Figure 7B). Also, the overall recognition rate reached a peak (81.5%) when the sample proportions for fist, spherical, and cylindrical grasping were ~30, ~30, and ~40%, respectively (Figure 7C). However, when the gesture sample in grasping training set was assigned uniformly (i.e., 17 sample for fist, spherical, or cylindrical grasping), the overall recognition rate was 80.8% (Table 2, last row).

**Figure 8:**
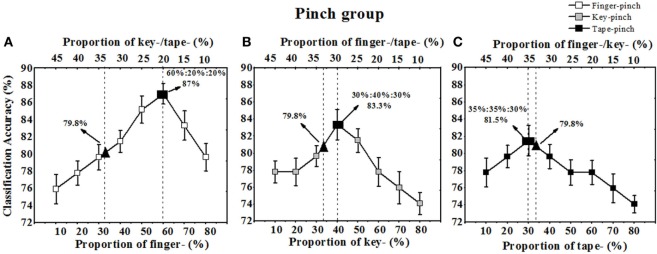
The total recognition rates of finger-, key-, and tape-grasping gesture varied with the sample proportion. **(A)** The overall Acc. varied with the sample proportion of finger pinch; **(B)** the overall Acc. varied with the sample proportion of key pinch; **(C)** the overall Acc. varied with the sample proportion of tape pinch, ▴ indicating the Acc. when sample proportion of finger-, key-, and tape-pinch gesture were one third, ■ indicating the peak Acc. when sample proportion was optimized in grasping training set.

For the pinch gesture group, similar trend of the overall recognition rate was observed. When the sample proportion for finger pinch varied from ~10 to ~80%, the peak overall recognition rate (87%) was obtained when the sample proportion for finger-pinch was ~60%, whereas the sample proportions for key, tape, and finger pinch were ~20% (see Figure 8A). A peak in the overall recognition rate (83.3%) also occurred when the training sample proportions for finger, key, and tape pinch were ~30, ~40, and ~30%, respectively (Figure 8B). Another peak in the overall recognition rate (81.5%) occurred when the training sample proportions for finger, key, and tape pinch were ~35, ~35, and ~30%, respectively (Figure 8C). When we equally assigned the samples of pinch gestures in training set (i.e., 17 sample for finger, key, or tape pinch) (Table 3, last row), the overall recognition rate was only 79.8%.

### Gesture Sample Proportion in the Training Set Affects the Inter-Class Distance in Feature Space

**Figure 9:**
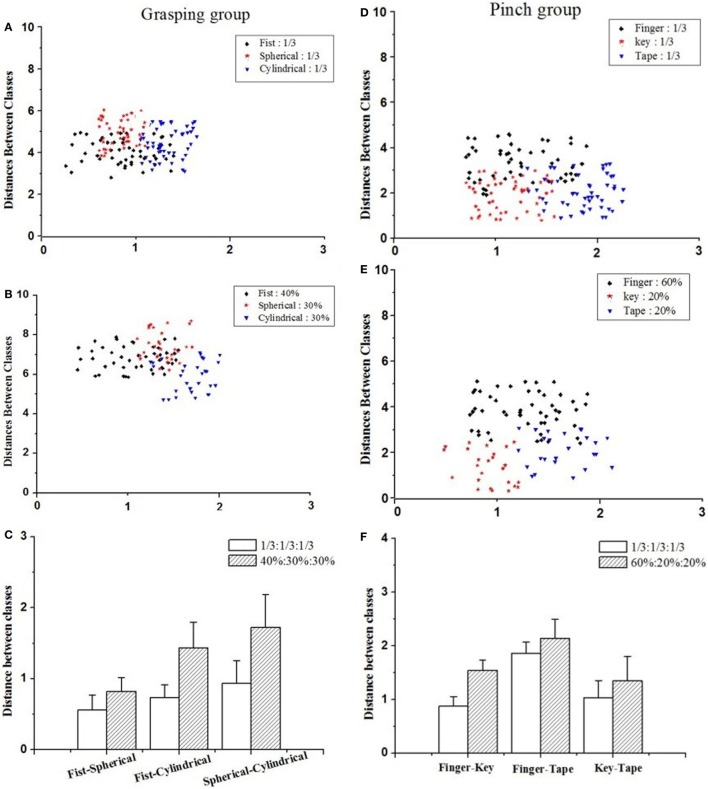
The distance between classes in feature space of grasping-gesture group and pinch gesture; **(A)** cluster analysis plot when sample of fist-, cylindrical- and spherical-grasping gestures was one third; **(B)** cluster analysis plot when sample of fist-, cylindrical- and spherical-grasping gestures was 40, 30, and 30%; **(C)** the comparison of *D*_out_ values for grasping group gestures; **(D)** cluster analysis plot when sample of finger-, key-, and tape-pinch gestures was one third; **(E)** cluster analysis plot when sample of finger-, key-, and tape-pinch gestures was 60, 20, and 20%; **(F)** the comparison of *D*_out_ values for pinch group gestures.

The sEMG feature vector of a gesture can be considered as a cluster to be classified among candidate hand gestures (see Figure 9). To determine how the sample proportion affects the recognition rate of hand gestures, we assessed the discrimination of the sEMG feature vector in feature space with the Mahalanobis distance between hand gesture classes (*D*_out_). We compared the *D*_out_ values of any two gestures in the grasping group (i.e., fist grasping vs. cylindrical grasping, fist grasping vs. spherical grasping, and cylindrical grasping vs. spherical grasping), and pinch group (i.e., finger pinch vs. key pinch, finger pinch vs. tape pinch, and key pinch vs. tape pinch).

As shown in Figures 9C,F, sample proportion assignment change in training set can significantly affect the Mahalanobis distance between any two hand gestures (*D*_out_). In the grasping group, when the sample of fist-, cylindrical-, and spherical-grasping gestures was conventionally set as one third, the *D*_out_ values for fist-cylindrical, fist-spherical, and cylindrical-spherical gestures were 0.5601 ± 0.21, 0.7347 ± 0.18, and 0.9366 ± 0.15, respectively. The corresponding overall recognition rate was 80.8% (see Figure 7). However, if the samples of fist-, cylindrical-, and spherical-grasping gestures were assigned as 40, 30, and 30%, the *D*_out_ values for the fist-cylindrical, fist-spherical, and cylindrical-spherical gestures were extended to 0.8252 ± 0.19, 1.4374 ± 0.31, and 1.7255 ± 0.46, respectively. The corresponding overall recognition rate was 83.3% (see Figure 7A). The Mahalanobis distances of the fist-cylindrical gestures or spherical-cylindrical gestures were nearly twofold enlarged. Similarly, in the pinch group, when the sample of finger-, key-, and tape-pinch gestures was conventionally set as one third, the *D*_out_ values for finger-key, finger-tape, and key-tape gestures were 0.8753 ± 0.18, 1.8635 ± 0.21, and 1.0353 ± 0.32, respectively. If the samples of finger-, key-, and tape-pinch gestures were assigned as 60, 20, and 20%, the *D*_out_ values for finger-key, finger-tape, and key-tape were extended to 1.5461 ± 0.19, 2.1367 ± 0.36, and 1.3468 ± 0.46, respectively. The results of paired-samples *t*-test (SPSS for Windows 13.0) indicated that, the Mahalanobis distance of finger-key gestures was significantly (*p* < 0.05) improved near twofold as much.

## Discussion

EMG-controlled robotic hand exoskeleton has been proposed to train paretic hand after stroke (Leonardis et al., 2015). Evidences indicate that the simultaneous movement of both non-paretic hand and paretic hand improve the neuro-muscular system to regain some stability and improve usage of the impaired limb (McCombe Waller and Whitall, 2008). Grasping and pinch is the most common hand movement with different finger coordination patterns. This study recorded the sEMG signals from forearm hand muscles through grasping or pinch tasks dominated with similar muscle activities, and the gesture-related myoelectric feature vector was set up with the ZC and WAMP of the IMF components after the EMD decomposition of sEMG. The impact of the gesture sample proportion in the training set on the gesture recognition efficiency was assessed, and our preliminarily results revealed that the recognition rate of alike hand gestures can be improved by optimizing the sample proportion due to the weight or impact of a gesture in a candidate gesture group.

Although the impact of sample size or constitution in training set on recognition efficiency have been observed in hand pattern recognition (Fratini et al., 2015), medical image classification (Wigdahl et al., 2013b), and human limb gesture identification (Chen et al., 2016a), this is the first time to quantify how the sample proportion of a candidate hand gesture influence its classification accuracy. Our results indicated that, for any grasping gesture or pinch gesture, the recognition rate of a hand gesture can be improved by increase the sample proportion of corresponding gesture in the training set. As shown in Figures 5 and 6, the recognition rate of a single gesture quickly improved when corresponding sample proportion increased in the training set. In fact, increasing the sample proportion of a gesture in training set implied enhancing the weight of this gesture in the training stage, and the classifier learned much more from this gesture. Therefore, as a result the improved recognition of interested gesture can be obtained. On other hand, the recognition rate of a gesture would decrease when the sample proportion reduced in the training set as well (in Figures 5 and 6, lower part). Our results revealed that the recognition of one gesture can be improved by increasing the weight of this gesture in the training set.

Unlike the proportional allocation of sample for classifier training, the present work affirmed that non-uniform sample assignment in training set may significantly improves those similar hand gestures recognition. In other words, each candidate grasping gesture or pinch gestures has different impact on classifier, however, it is usually assumed that each class has an equal *a priori* probability of occurrence and the same number of samples for each class had been conventionally allocated in the training set. In fact, as shown in Figures 5 and 6, although the classification accuracies of each grasping gesture or pinch gesture would increase with its sample proportion improved and decrease with its sample proportion dropped, our study also show the slope of the curve is different for each gesture. For example, the classification accuracies of finger-pinch dropped faster than that of key pinch or tape pinch when sample proportion decreased (Figures 6B,C). Then, we can assume that the sample proportion of finger-pinch task gave rise to more impact on the recognition of pinch gestures, and more finger-pinch samples is help to get higher classification accuracies. Thus, assigning more finger-pinch samples (60%) in training set is expected for the higher recognition rate (87%) of similar pinch gestures.

Although a classifier trained with equally assigned samples is enough for recognition of hand gestures with distinguished muscle activity patterns (Urwyler et al., 2015), there remain obstacles for similar hand gestures recognition due to the similar muscle activity patterns and similar sEMG features (Geng et al., 2014). As the tasks tested in our study, grasping gestures or pinch gestures requires similar muscular contraction pattern, which makes it challenging to discriminate the characteristic vector of similar gestures in the feature space. As illustrated in Figure 9A, the distance between classes for the gestures in the grasping group are too short to be distinguished, however, when the classifier has been trained with unequally assigned samples of candidate grasping or pinch gestures, the Mahalanobis distance for these similar gestures is significantly enlarged. Then, the classification accuracy is improved as well. Furthermore, many more factors should be considered when we assess the overall recognition rate for candidate gestures, such as the slope of ascending recognition rate with increasing sample proportion and the slope of descending recognition rates with decreasing sample proportion. For a training set with constant sample size investigated in present study, the sample proportion should be carefully selected. As shown in Figures 7 and 8, when an appropriate assignment of the sample proportion in the training set for grasping gestures and pinch gestures, the highest overall recognition rate was obtained for similar grasping or pinch gestures.

To the best of our knowledge, the present work is the first to evaluate the effect of the sample proportion in the training set on the recognition rate of hand gestures, and the classification accuracy of similar grasping or pinch gestures can be improved by unequally assigning the samples in training set. Then, an alternative way improving the classification efficiency is to optimize the sample proportion of candidate patterns in training set due to corresponding impact of a pattern on the recognition rate. In other words, we can assign more samples of the candidate gesture with higher weight to obtain better recognition, however, these preliminary results just give a clue that the weight of candidate gesture may be different, and sample proportion in training set should be optimized for improving classification. Further studies are needed to explore how to set the optimal sample proportion in training set, and the classifier will be improved as well (Pratama et al., 2016; Lughofer et al., 2017; Rubio, 2017a,b). On other hand, enlarging the sample number of one candidate gesture may induce overfitting or overlearning in classifier, it can be suggested to compare the slopes of sample proportion vs. Acc. curve among the candidate gestures (see Figure 5 or Figure 6), and focused on the sample proportion allocated, the quickly ascending and descending part of sample proportion vs. Acc. curves. For the bimanual rehabilitation after stroke with robotic hand exoskeleton, both the gesture and force for hand movement should be implemented to paretic hand training. Also, muscle activation can be act as a good reference guide in bilateral training (McCombe Waller et al., 2006), especially the grasping or pinch force can be estimated with sEMG of non-paretic hand and then replicated as robotic assistance for the paretic hand (Leonardis et al., 2015). In addition to recognition of hand gesture, the finger force or finger joint of non-paretic hand dominated with similar muscle activities will be estimated by sEMG recording next.

## Author Contributions

YZ and ZG collected the data; ZY analyzed the data. WH, YL, XW, and XZ designed the work. WH drafted the work. WH and GL interpreted the data. LC helped to revise the manuscript. WH and QX created the final report.

## Conflict of Interest Statement

The authors declare that the research was conducted in the absence of any commercial or financial relationships that could be construed as a potential conflict of interest.

## References

[B1] AbdelMaseeh M. Chen T.-W. Stashuk D. W. (2016). Extraction and classification of multichannel electromyographic activation trajectories for hand movement recognition. IEEE Trans. Neural Syst. Rehabil. Eng. 24, 662–673. 10.1109/TNSRE.2015.2447217 26099148

[B2] Al-Angari H. M. Kanitz G. Tarantino S. Cipriani C. (2016). Distance and mutual information methods for EMG feature and channel subset selection for classification of hand movements. Biomed. Signal Process. Control 27, 24–31. 10.1016/j.bspc.2016.01.011

[B3] Alba E. Garcia-Nieto J. Jourdan L. Talbi E. G. (2007). Gene selection in cancer classification using PSO/SVM and GA/SVM hybrid algorithms. IEEE Congress Evol. Comput. 4, 284–290. 10.1109/CEC.2007.4424483

[B4] Amsuess S. Goebel P. Graimann B. Farina D. (2015). A multi-class proportional myocontrol algorithm for upper limb prosthesis control: validation in real-life scenarios on amputees. IEEE Trans. Neural Syst. Rehabil. Eng. 23, 827–836. 10.1109/TNSRE.2014.2361478 25296406

[B5] Castro M. C. Arjunan S. P. Kumar D. K. (2015). Selection of suitable hand gestures for reliable myoelectric human computer interface. Biomed. Eng. Online 14, 30. 10.1186/s12938-015-0025-5 25889735 PMC4393867

[B6] Castro M. C. F. Colombini E. L. Aquino P. T. Arjunan S. P. Kumar D. K. (2014). sEMG feature evaluation for identification of elbow angle resolution in graded arm movement. Biomed. Eng. Online 13, 155. 10.1186/1475-925X-13-155 25422006 PMC4280697

[B7] Cauraugh J. H. Lodha N. Naik S. K. Summers J. J. (2010). Bilateral movement training and stroke motor recovery progress: a structured review and meta-analysis. Hum. Mov. Sci. 29, 853–870. 10.1016/j.humov.2009.09.004 19926154 PMC2889142

[B8] Chen J. Chen X. L. Yang J. Shan S. G. Wang R. P. Gao W. (2009). Optimization of a training set for more robust face detection. Pattern Recognit. 42, 2828–2840. 10.1016/j.patcog.2009.02.006

[B9] Chen X. M. Pun S. H. Zhao J. F. Mak P. U. Liang B. D. Vai M. I. (2016a). Effects of human limb gestures on galvanic coupling intra-body communication for advanced healthcare system. Biomed. Eng. Online 15, 60 10.1186/s12938-016-0192-z 27230849 PMC4882836

[B10] Chen X. M. Pun S. H. Zhao J. F. Mak P. U. Liang B. D. Vai M. I. (2016b). Effects of human limb gestures on galvanic coupling intra-body communication for advanced healthcare system. Biomed. Eng. Online 15, 60 10.1186/s12938-016-0192-z 27230849 PMC4882836

[B11] Foody G. M. Mcculloch M. B. Yates W. B. (1995). The effect of training set size and composition on artificial neural network classification. Int. J. Remote Sens. 16, 1707–1723. 10.1080/01431169508954396

[B12] Francini A. Romeo S. Cifelli M. Gori D. Domenici V. Sebastiani L. (2017). H-1 NMR and PCA-based analysis revealed variety dependent changes in phenolic contents of apple fruit after drying. Food Chem. 221, 1206–1213. 10.1016/j.foodchem.2016.11.038 27979079

[B13] Fratini A. Sansone M. Bifulco P. Cesarelli M. (2015). Individual identification via electrocardiogram analysis. Biomed. Eng. Online 14, 78 10.1186/s12938-015-0072-y 26272456 PMC4535678

[B14] Geng Y. J. Zhang X. F. Zhang Y. T. Li G. L. (2014). A novel channel selection method for multiple motion classification using high-density electromyography. Biomed. Eng. Online 13, 102. 10.1186/1475-925X-13-102 25060509 PMC4125347

[B15] Hong T. Zhang X. Ma H. Chen Y. Chen X. (2016). Fatiguing effects on the multi-scale entropy of surface electromyography in children with cerebral palsy. Entropy 18, 177 10.3390/e18050177

[B16] Jain A. K. Duin R. P. W. Mao J. C. (2000). Statistical pattern recognition: a review. IEEE Trans. Pattern Anal. Mach. Intell. 22, 4–37. 10.1109/34.824819

[B17] Khezri M. Jahed M. (2007). Real-time intelligent pattern recognition algorithm for surface EMG signals. Biomed. Eng. Online 6, 45. 10.1186/1475-925X-6-45 18053184 PMC2222669

[B18] Leonardis D. Barsotti M. Loconsole C. Solazzi M. Troncossi M. Mazzotti C. (2015). An EMG-controlled robotic hand exoskeleton for bilateral rehabilitation. IEEE Trans. Haptics 8, 140–151. 10.1109/TOH.2015.2417570 25838528

[B19] Li H. Q. Yuan D. Y. Ma X. D. Cui D. Y. Cao L. (2017). Genetic algorithm for the optimization of features and neural networks in ECG signals classification. Sci. Rep. 7, 41011. 10.1038/srep41011 28139677 PMC5282533

[B20] Lima C. A. M. Coelho A. L. V. Madeo R. C. B. Peres S. M. (2016). Classification of electromyography signals using relevance vector machines and fractal dimension. Neural Comput. Appl. 27, 791–804. 10.1007/s00521-015-1953-5

[B21] Liu J. Li X. Y. Li G. L. Zhou P. (2014). EMG feature assessment for myoelectric pattern recognition and channel selection: a study with incomplete spinal cord injury. Med. Eng. Phys. 36, 975–980. 10.1016/j.medengphy.2014.04.003 24844608 PMC4043864

[B22] Lorrain T. Jiang N. Farina D. (2011). Influence of the training set on the accuracy of surface EMG classification in dynamic contractions for the control of multifunction prostheses. J. Neuroeng. Rehabil. 8, 25. 10.1186/1743-0003-8-25 21554700 PMC3113948

[B23] Luft A. R. Mccombewaller S. Whitall J. Forrester L. W. Macko R. Sorkin J. D. (2005). Repetitive bilateral arm training and motor cortex activation in chronic stroke: a randomized controlled trial. JAMA 292, 1853–1861. 10.1001/jama.292.15.1853 PMC2930817 15494583

[B24] Lughofer E. Pratama M. Skrjanc I. (2017). Incremental rule splitting in generalized evolving fuzzy systems for autonomous drift compensation. IEEE Transact. Fuzzy Syst. 99, 1 10.1109/TFUZZ.2017.2753727

[B25] Marchetti M. Onorati F. Matteucci M. Mainardi L. Piccione F. Silvoni S. (2013). Improving the efficacy of ERP-based BCIs using different modalities of covert visuospatial attention and a genetic algorithm-based classifier. PLoS ONE 8:e53946. 10.1371/journal.pone.0053946 23342043 PMC3544767

[B26] Martins M. Costa L. Frizera A. Ceres R. Santos C. (2014). Hybridization between multi-objective genetic algorithm and support vector machine for feature selection in walker-assisted gait. Comput. Met. Programs Biomed. 113, 736–748. 10.1016/j.cmpb.2013.12.005 24444751

[B27] McCombe Waller S. Harris-Love M. Liu W. Whitall J. (2006). Temporal coordination of the arms during bilateral simultaneous and sequential movements in patients with chronic hemiparesis. Exp. Brain Res. 168, 450–454. 10.1007/s00221-005-0235-3 16331507

[B28] McCombe Waller S. Whitall J. (2008). Bilateral arm training: why and who benefits? NeuroRehabilitation 23, 29–41. 18356587 PMC2953420

[B29] Oujamaa L. Relave I. Froger J. Mottet D. Pelissier J. Y. (2009). Rehabilitation of arm function after stroke. Literature review. Ann. Phys. Rehabil. Med. 52, 269–293. 10.1016/j.rehab.2008.10.003 19398398

[B30] Peerdeman B. Boere D. Witteveen H. In’t Veld R. H. Hermens H. Stramigioli S. (2011). Myoelectric forearm prostheses: state of the art from a user-centered perspective. J. Rehabil. Res. Dev. 48, 719–737. 10.1682/JRRD.2010.08.0161 21938658

[B31] Phinyomark A. Hu H. Phukpattaranont P. Limsakul C. (2012). Application of linear discriminant analysis in dimensionality reduction for hand motion classification. Meas. Sci. Rev. 12, 82–89. 10.2478/v10048-012-0015-8

[B32] Pratama M. Lughofer E. Meng J. E. Anavatti S. Lim C. P. (2016). Data driven modelling based on recurrent interval-valued metacognitive scaffolding fuzzy neural network. Neurocomputing 262, 4–27. 10.1016/j.neucom.2016.10.093

[B33] Rubio J. D. J. (2017a). A method with neural networks for the classification of fruits and vegetables. Soft Comput. 21, 7207–7220. 10.1007/s00500-016-2263-2

[B34] Rubio J. D. J. (2017b). Stable Kalman filter and neural network for the chaotic systems identification. J. Franklin Inst. 354, 7444–7462. 10.1016/j.jfranklin.2017.08.038

[B35] Sapsanis C. Georgoulas G. Tzes A. Lymberopoulos D. (2013). Improving EMG based classification of basic hand movements using EMD. Conf. Proc. IEEE Eng. Med. Biol. Soc. 2013, 5754–5757. 10.1109/EMBC.2013.6610858 24111045

[B36] Serdio F. Lughofer E. Zavoianua A. C. Pichler K. Pichler M. Buchegger T. (2017). Improved fault detection employing hybrid memetic fuzzy modeling and adaptive filters. Appl. Soft Comput. 51, 60–82. 10.1016/j.asoc.2016.11.038

[B37] Shang X. J. Tian Y. T. Li Y. (2011). Feature extraction and classification of sEMG based on ICA and EMD decomposition of AR model. Int Conf. Electron. Commun. Control (Icecc) 1464–1467. 10.1109/ICECC.2011.6067702

[B38] Urwyler P. Rampa L. Stucki R. Buchler M. Muri R. Mosimann U. P. (2015). Recognition of activities of daily living in healthy subjects using two ad-hoc classifiers. Biomed. Eng. Online 14, 54. 10.1186/s12938-015-0050-4 26048452 PMC4457983

[B39] Wigdahl J. Agurto C. Murray V. Barriga S. Soliz P. (2013a). Training set optimization and classifier performance in a top-down diabetic retinopathy screening system. Med. Imaging Comput. Aided Diagn. 8670 10.1117/12.2007931

[B40] Wigdahl J. Murray V. Barriga S. Soliz P. (2013b). Training set optimization and classifier performance in a top-down diabetic retinopathy screening system. SPIE Med. Imag. 8670 10.1117/12.2007931

[B41] Windrich M. Grimmer M. Christ O. Rinderknecht S. Beckerle P. (2016). Active lower limb prosthetics: a systematic review of design issues and solutions. Biomed. Eng. Online 15, 140. 10.1186/s12938-016-0284-9 28105948 PMC5249019

[B42] Young A. J. Hargrove L. J. Kuiken T. A. (2012). Improving myoelectric pattern recognition robustness to electrode shift by changing interelectrode distance and electrode configuration. IEEE Trans. Biomed. Eng. 59, 645–652. 10.1109/TBME.2011.2177662 22147289 PMC4234037

[B43] Zhang Y. Hou W. Luo H. Wu X. Liao Y. Fan X. (2016). The impact of sEMG feature weight on the recognition of similar grasping gesture. IEEE Int. Conf. Adv. Robot. Mechatronics 260–265. 10.1109/ICARM.2016.7606929

